# Co-administration of human MSC overexpressing HIF-1α increases human CD34^+^ cell engraftment in vivo

**DOI:** 10.1186/s13287-021-02669-z

**Published:** 2021-12-07

**Authors:** Silvia Preciado, Mª Salomé Sirerol-Piquer, Sandra Muntión, Lika Osugui, Gerardo J. Martí-Chillón, Almudena Navarro-Bailón, Pilar Sepúlveda, Fermín Sánchez-Guijo

**Affiliations:** 1 Cell Therapy Unit, Hematology Department, University Hospital of Salamanca, IBSAL, University of Salamanca, Paseo de San Vicente 58-182, 37007 Salamanca, Spain; 2 Departamento de Biología Celular, Biología Funcional y Antropología Física, University of Valencia, Burjassot, Spain; 3 Instituto de Biotecnología y Biomedicina (BioTecMed), University of Valencia, Burjassot, Spain; 4 Regenerative Medicine and Heart Transplantation Unit, Instituto de Investigación Sanitaria La Fe, Valencia, Spain; 5 RETIC TerCel, ISCIII, Madrid, Spain; 6 Centro en Red de Medicina Regenerativa y Terapia Celular de Castilla y León, Salamanca, Spain

**Keywords:** Engraftment, HIF-1α, Stem cell transplantation, Mesenchymal stromal cells, Hematopoietic stem cells, Graft failure, Poor graft function

## Abstract

**Background:**

Poor graft function or graft failure after allogeneic stem cell transplantation is an unmet medical need, in which mesenchymal stromal cells (MSC) constitute an attractive potential therapeutic approach. Hypoxia-inducible factor-1α (HIF-1α) overexpression in MSC (HIF-MSC) potentiates the angiogenic and immunomodulatory properties of these cells, so we hypothesized that co-transplantation of MSC-HIF with CD34^+^ human cord blood cells would also enhance hematopoietic stem cell engraftment and function both in vitro and in vivo.

**Methods:**

Human MSC were obtained from dental pulp. Lentiviral overexpression of HIF-1α was performed transducing cells with pWPI-green fluorescent protein (GFP) (MSC WT) or pWPI-HIF-1α-GFP (HIF-MSC) expression vectors. Human cord blood CD34^+^ cells were co-cultured with MSC WT or HIF-MSC (4:1) for 72 h. Then, viability (Annexin V and 7-AAD), cell cycle, ROS expression and immunophenotyping of key molecules involved in engraftment (CXCR4, CD34, ITGA4, c-KIT) were evaluated by flow cytometry in CD34^+^ cells. In addition, CD34^+^ cells clonal expansion was analyzed by clonogenic assays. Finally, in vivo engraftment was measured by flow cytometry 4-weeks after CD34^+^ cell transplantation with or without intrabone MSC WT or HIF-MSC in NOD/SCID mice.

**Results:**

We did not observe significant differences in viability, cell cycle and ROS expression between CD34^+^ cells co-cultured with MSC WT or HIF-MSC. Nevertheless, a significant increase in CD34, CXCR4 and ITGA4 expression (*p* = 0.009; *p* = 0.001; *p* = 0.013, respectively) was observed in CD34^+^ cells co-cultured with HIF-MSC compared to MSC WT. In addition, CD34^+^ cells cultured with HIF-MSC displayed a higher CFU-GM clonogenic potential than those cultured with MSC WT (*p* = 0.048). We also observed a significant increase in CD34^+^ cells engraftment ability when they were co-transplanted with HIF-MSC compared to CD34^+^ co-transplanted with MSC WT (*p* = 0.016) or alone (*p* = 0.015) in both the injected and contralateral femurs (*p* = 0.024, *p* = 0.008 respectively).

**Conclusions:**

Co-transplantation of human CD34^+^ cells with HIF-MSC enhances cell engraftment in vivo. This is probably due to the ability of HIF-MSC to increase clonogenic capacity of hematopoietic cells and to induce the expression of adhesion molecules involved in graft survival in the hematopoietic niche.

**Supplementary Information:**

The online version contains supplementary material available at 10.1186/s13287-021-02669-z.

## Background

Allogeneic hematopoietic stem cell transplantation (allo-HSCT) remains as the only curative therapeutic approach for a variety of hematological diseases. An adequate hematopoietic function after an allo-HSCT is not only dependent on the number of hematopoietic stem cells (HSC) infused that is clearly an important factor for engraftment, but also on the functioning of the bone marrow (BM) microenvironment, a key regulator of the hematopoietic function. The latter is often damaged in this setting [1, 2]. Among the cell types in the BM microenvironment, probably Mesenchymal Stromal Cells (MSC) have attracted most of the attention for a number of reasons, including their regenerative potential within the BM niche and their immunomodulatory properties [3].

These properties have prompted their assessment in vivo for the treatment of important complications after allo-HSCT, especially graft-versus-host disease (GVHD) or poor graft function [4–6]. Human MSC can be isolated from several sources although the most common are BM and adipose tissue. Dental MSC (dental pulp, apical papilla, dental follicle and periodontal ligament) are also considered an attractive source of MSC due to their easy accessibility and in vitro expansion and their low immunogenicity [7]. We and others have shown that MSC are able to maintain in vitro hematopoiesis and that MSC co-infusion may modulate host alloreactivity and promote better engraftment of donor HSC [8, 9]. Some preliminary clinical experiences of the use of MSC to improve hematopoietic engraftment after allo-SCT [10, 11] are encouraging, but new strategies boosting these effects are needed.

In this regard, it has been described that preconditioning MSC with hypoxia or the overexpression of hypoxia-inducible factor-1α (HIF-1α) potentiates the therapeutic properties of MSC [12–15]. HIF-1α is a transcription factor that is activated in response to low oxygen levels, enabling cell adaptation to hypoxia modifying cell metabolism and cell survival. HIF-1α stabilization contributes to maintaining the undifferentiated and multipotent status of MSC [16], induces the activation of Notch and Akt signaling pathways, which regulate survival, migration and proliferation [17, 18], and activates the production of angiogenic factors [19].

We have previously demonstrated that the overexpression of HIF-1α in MSC improves migration, proliferation capacity, angiogenic and immunomodulatory properties of these cells leading that in turns potentiates their regenerative capacity in experimental models of ischemia [20–22]. Others reported that co-culture of human fetal liver stromal cells expressing HIF-1α improved the maintenance of self-renewal and pluripotency of human embryonic stem cells [23]. Indeed, MSC depend on HIF factors for maintenance and regulation of hematopoiesis [14].

In the current work, we show the ability of HIF-MSC to promote hematopoietic stem cell function and engraftment both in vitro and in vivo.

## Methods

### Cell culture and lentiviral transduction

Human MSC obtained from dental pulp were purchased from Inbiomed (Inbiobank, San Sebastian, Spain). Cells were cultured in Dulbecco’s modified Eagle’s medium (DMEM)-low glucose (Gibco, Life Technologies) supplemented with 10% fetal bovine serum (FBS; Gibco, Life Technologies) and 1% penicillin/streptomycin (Gibco) in a humidified atmosphere of 95% air and 5% CO_2_ at 37ºC.

Overexpression of HIF-1α was performed as previously described [22]. Briefly, MSC were transduced with a control pWPI-green fluorescent protein lentivirus (MSC wild type or MSC WT) or a HIF-1α-overexpressing pWPI-HIF-1α-GFP lentivirus (HIF-MSC) (http://addgene.org cat. #12254) daily for 3 days. Transduction efficiency was evaluated by flow cytometry (Coulter EPICS XL flow cytometer; Beckman Coulter) to determine the percentage of GFP-positive cells. The percentages of infection obtained were higher than 90% in all cases (Additional file 2: Figure S1). Cells were expanded and then cryopreserved until used.

For immunophenotypic characterization, MSC were incubated with the corresponding monoclonal antibodies (see Additional file 1). Then, they were acquired on a FACS Canto II device (BD Biosciences San Jose, CA) using the FACSDiva 6.1 software (BD Biosciences) and analyzed with the Infinicyt software.

### CD34^+^ cell isolation

CD34^+^ cells were isolated from 24 fresh umbilical cord blood (UCB) units obtained in the Obstetrics Department of the University Hospital of Salamanca following standard procedures, after informed consent was obtained from the corresponding mothers (range 27–40 years). Mononucleated cells were isolated by Ficoll-Paque density gradient centrifugation, and then, CD34^+^ cells were purified by immunomagnetic sorting in an AutoMACS (MiltenyiBiotec GmbH) after labeling with the human CD34 MicroBead Kit (MiltenyiBiotec) according to the manufacturer’s recommendations. Purity of cells was confirmed by flow cytometry using FITC-CD34 (eBioscience), and the viability was evaluated by labeling cells with 7-amino-actinomycin D (7-AAD). Purity was superior to 90% in all cases and their viability was higher than 85%. All experimental procedures were approved by local Ethics Committee (code PI26/03/2018).

### Co-culture of UCB CD34^+^ cells with MSC

For the in vitro experiments indicated below, 2 × 10^5^ CD34^+^ cells from 10 UCB samples were incubated either with 5 × 10^4^ MSC WT or HIF-MSC. Cultures were maintained for 72 h in a volume of 2.5 ml Roswell Park Memorial Institute Medium (RPMI; Gibco, Life Technologies) per well at 37ºC and 5% of CO_2_ (normoxic conditions) in a humidified atmosphere.

### Apoptosis assays and cell cycle analysis

After 72 h of co-culture, CD34^+^ cells were collected and washed. For apoptosis assays, cells were stained with Annexin V and 7-AAD using the BD Pharmigen PE Annexin V Apoptosis Detection Kit I (BD Biosciences). For cell cycle analysis, cells were stained with propidium iodide using the kit FxCycletm PI/RNase Staining Solution (Life Technologies), according to manufacturer’s instructions. Samples were acquired on a FACS Canto II device (BD Biosciences San Jose, CA) using the FACSDiva 6.1 software (BD Biosciences). At least 1.5 × 10^5^ events were recorded. For apoptosis assays, data were analyzed using Infinicyt (Cytognos). Cells were classified as early apoptotic, late apoptosis or dead if they were Annexin V^+^/7-AAD^−^, Annexin V^+^/7-AAD^+^ or Annexin V^−^/7-AAD^+^, respectively, as previously reported [2]. Ten samples were used for these experiments. For cell cycle analysis, ModFit LT version 5.0.9 (Verity Software) was used. Seven samples were analyzed. Data are represented as percentage of cells in each cell cycle phase.

### Intracellular reactive oxygen species (ROS)

Intracellular ROS expression was assessed in both experimental groups as described [24]. Briefly, CD34^+^ cells suspended in phosphate-buffered saline (PBS; Gibco) were stained with the cell-permeant probe 2,7-dichlorodihydrofluorescein diacetate (DCFH-DA; Sigma-Aldrich, St. Louis, MO, USA) at a final concentration of 10 µmol/l, at 37 °C in the dark for 30 min. Then, cells were labeled with 7-AAD and acquired on a FACSCanto II flow cytometer (BD Biosciences San Jose, CA) using the FACSDiva 6.1 software (BD Biosciences). Experiments were performed in ten samples. Data are represented as median fluorescence intensity (MFI).

### Flow cytometric analysis of proteins involved in cell engraftment and hematopoiesis

CD34^+^ cells from 10 samples were collected after the co-culture with MSC WT or HIF-MSC, washed with PBS and incubated with the corresponding monoclonal antibodies (see Additional file 1). Then, they were acquired on a FACS Canto II device (BD Biosciences San Jose, CA) using the FACSDiva 6.1 software (BD Biosciences) and analyzed with the Infinicyt software. Data were represented as MFI.

### Clonogenic assays

Clonal growth of CD34^+^ cells was evaluated after 72 h of co-culture with MSC WT or HIF-MSC. For this purpose, CD34^+^ cells were recovered and 2,500 cells were seeded in methylcellulose Stem MACS Media (HSC-CFU complete without Epo, human) (Miltenyi Biotec, Germany) that contains stem cell factor (SCF), granulomonocytic colony-stimulating factor (GM-CSF), granulocytic colony-stimulating factor (G-CSF), interleukin-3 and interleukin-6, to quantify progenitor cell colony-forming unit granulocyte/macrophage (CFU-GM), according to the manufacturer’s instructions. After 14 days at 37ºC in a fully humidified atmosphere of 5% CO_2_, colonies were scored using an inverted microscope. Ten experiments were performed.

### Analysis of human hematopoietic engraftment in mice

For the in vivo studies, 6-week-old nonobese diabetic/severe combined immunodeficient mice (NOD/SCID; NOD.CB17-Prkdcscid/NcrCrl) were purchased from Charles River Laboratories (Barcelona, Spain) and maintained in the Animal Care Facility of the University of Salamanca. All procedures followed the Spanish and European Union guidelines (RD 1201/05 and 86/609/CEE) and were approved by the Bioethics Committee of the University of Salamanca (reg. 201100007924). The murine xenotransplant model was established as previously reported, with minor modifications [9, 25]. Thirty-six female mice were used establishing three experimental groups: (1) 1 × 10^5^ UCB CD34^+^ cells, n = 8; (2) 1 × 10^5^ UCB CD34^+^ cells + 5 × 10^5^ MSC WT, n = 13; and (3) 1 × 10^5^ UCB CD34^+^ cells + 5 × 10^5^ HIF-MSC, n = 15. Before UCB transplantation, mice were irradiated with 350 cGy of total body irradiation using a cesium source (Gammacell-200, Nordion International). Then, animals were anesthetized with a mixture of ketamine (90 mg/kg; Imalgene 500) and xylazine (10 mg/kg; Rompun 2%, KVP Pharma) before MSC administration via intrafemorally, through the knee in a well-established method, described in prior works from our group and others [9, 25–27]. Four hours after MSC administration, CD34^+^ cells were injected intravenously. Finally, four weeks after transplantation, mice were killed, and hematopoietic engraftment was evaluated in the injected (right) and contralateral (left) femurs and spleen by flow cytometry. The information on the monoclonal antibodies and analysis is detailed in Additional file 1.

### Statistical analysis

Values are summarized as median and range. The nonparametric Wilcoxon signed-rank test was used to compare the differences between paired results, and the nonparametric Mann–Whitney U test was used to compare the differences between unpaired results. Differences were considered to be statistically significant for values of *p* < 0.05. All statistical analyses were performed on the GraphPad Prism version 5.00 for Windows (GraphPad Software, San Diego, CA).

## Results

### Characterization of MSC WT and HIF-MSC

Both MSC WT and HIF-MSC displayed the characteristic fibroblastic-like morphology and were adherent to plastic surfaces. Immunophenotypic analysis showed that all samples expressed CD90, CD44, CD73, CD105 and CD166, while they were negative for hematopoietic markers (Additional file 3: Figure S2).

### HIF-MSC do not alter the viability and proliferative capacity of CD34^+^ cells

**Fig. 1:**
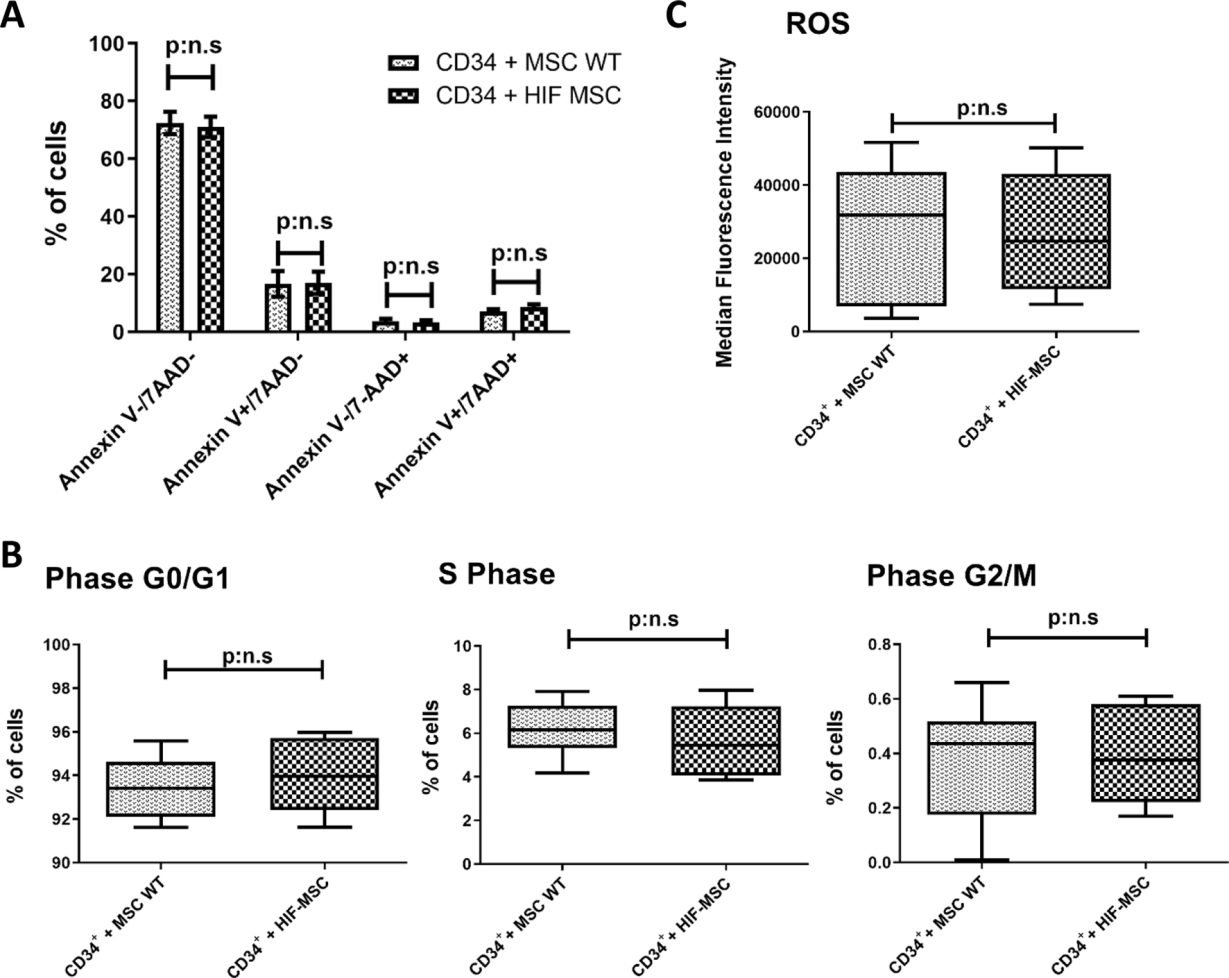
Viability, proliferation and ROS expression in CD34^+^ cells after co-culture with MSC WT or HIF-MSC. Apoptosis assays in CD34^+^ cells previously co-cultured with MSC WT or HIF-MSC during 72 h. CD34^+^ cells were incubated with Annexin V, 7-AAD and CD34, and the expression of different cell surface markers was analyzed by flow cytometry. Cells were classified as viable (Annexin V^−^/7-AAD^−^), early apoptotic (Annexin V^+^/7-AAD^−^), late apoptotic (Annexin V^+^/7-AAD^+^) or dead (Annexin V^−^/7-AAD^+^). Data expressed as mean of the percentage of cells in the different conditions (**A**). Cell cycle profiling of CD34^+^ cells after 72 h of culture in each condition analyzed by flow cytometry. Data are represented as mean of the percentage of cells in each phase (**B**). Intracellular reactive oxygen species (ROS) expression of each population represented as median of fluorescence intensity (**C**). Ten experiments were done for each group. p:n.s means *p* > 0.05

We set an in vitro co-culture experiment to evaluate the effect of HIF-MSC in CD34^+^ cells. Cell viability assays of CD34^+^ cells were performed after 72 h of co-culture with MSC (n = 10). 78.74% [60.64%—81.66%] of CD34^+^ cells were viable when co-cultured with MSC WT, with no significant differences with the co-culture with HIF-MSC (75.78% [62.61–77.75%]) (Annexin V^−^/7-AAD^−^). There were also no significant differences in the percentage of early apoptotic cells (Annexin V^+^/7-AAD^−^), late apoptotic cells (Annexin V^+^/7-AAD^+^) and dead cells (Annexin V^−^/7-AAD^+^) between both groups (Fig. 1A).

Regarding cell cycle, both CD34^+^ cells co-cultured with MSC WT or HIF-MSC show similar profiles. We did not find significant differences in the percentage of cells in Phase G0/G1 (93.42% [92.11–94.63%] vs 93.97% [92.41–95.72%]), Phase G2/M (0.43% [0.17–0.51%] vs 0.37% [0.22–0.58%]) and S Phase (6.15% [5.30–7.27%] vs 5.44% [40.6–7.22%]) between MSC WT and HIF-MSC, respectively (n = 7) (Fig. 1B).

### Intracellular ROS levels of CD34^+^ cells are not modified in co-culture with HIF-MSC

We also explored the possibility that HIF-MSC when co-cultured with CD34^+^ cells lowered their ROS, making them less vulnerable. When assessing the levels of intracellular ROS in CD34^+^ cells of both experimental conditions after 72 h of co-culture, we did not observe significant differences between them (n = 10) (Fig. 1C).

### Culture of CD34^+^ cells with HIF-MSC increases their expression of surface molecules involved in engraftment

**Fig. 2:**
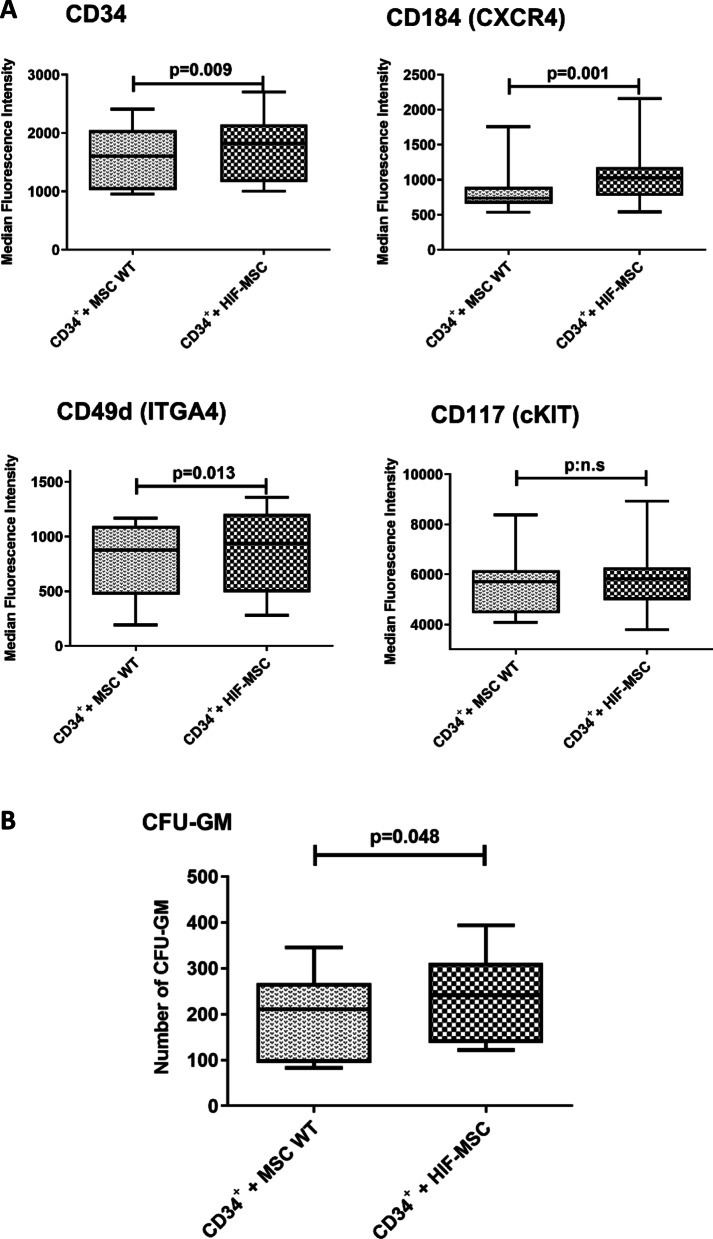
Expression of surface proteins involved in hematopoiesis and engraftment in CD34^+^ cells and clonogenic capacity. Median fluorescence intensity of different surface proteins involved in hematopoiesis maintenance and engraftment as CD34, CXCR4, ITGA-4 and c-KIT was evaluated by flow cytometry. Samples were acquired on a FACS Canto flow cytometer (**A**). Total CFU-GM from CD34^+^ cells were scored after 14 days in methylcellulose medium. CD34^+^ cells were cultured with MSC WT or HIF -MSC for 72 h, and then, 1,500 cells were seeded into methylcellulose medium (**B**). Data are represented as mean of 10 experiments for each group. p:n.s means *p* > 0.05

We next decided to test whether HIF-MSC when co-cultured with CD34^+^ cells could enhance their expression of proteins involved in hematopoietic engraftment and hematopoiesis maintenance. Surface expression in CD34^+^ cells of some key molecules involved in hematopoietic engraftment and hematopoiesis maintenance was also evaluated (n = 10). We found a significant increase in the expression of Hematopoietic Progenitor Cell Antigen (CD34), C-X-C Motif Chemokine Receptor 4 (CXCR4) and Integrin Subunit Alpha (ITGA4) in CD34^+^ cells that have been previously co-cultured with HIF-MSC compared to those co-cultured with MSC WT (*p* = 0.009, *p* = 0.001 and *p* = 0.013, respectively). We did not find significant changes in c-KIT expression (Fig. 2A).

### Culture of CD34^+^ cells with HIF-MSC increases their clonogenic capacity in vitro

Regarding the evaluation of in vitro functional effects induced after co-culture, we observed that the capacity of CD34^+^ cells to form CFU-GM colonies was significantly higher when they were co-cultured with HIF-MSC in comparison with MSC WT (*p* = 0.048). There were no differences in the shape or size of the colonies between both groups (Fig. 2B). Clonogenic studies were performed in ten samples. Our data together suggested that MSC-HIF enhance molecules engraftment expression and clonogenic capacity of CD34^+^ cells fostering the usage of both cell types for in vivo therapeutic purposes.

### Co-transplantation of CD34^+^ cells with HIF-MSC improves their engraftment ability in vivo

**Fig. 3:**
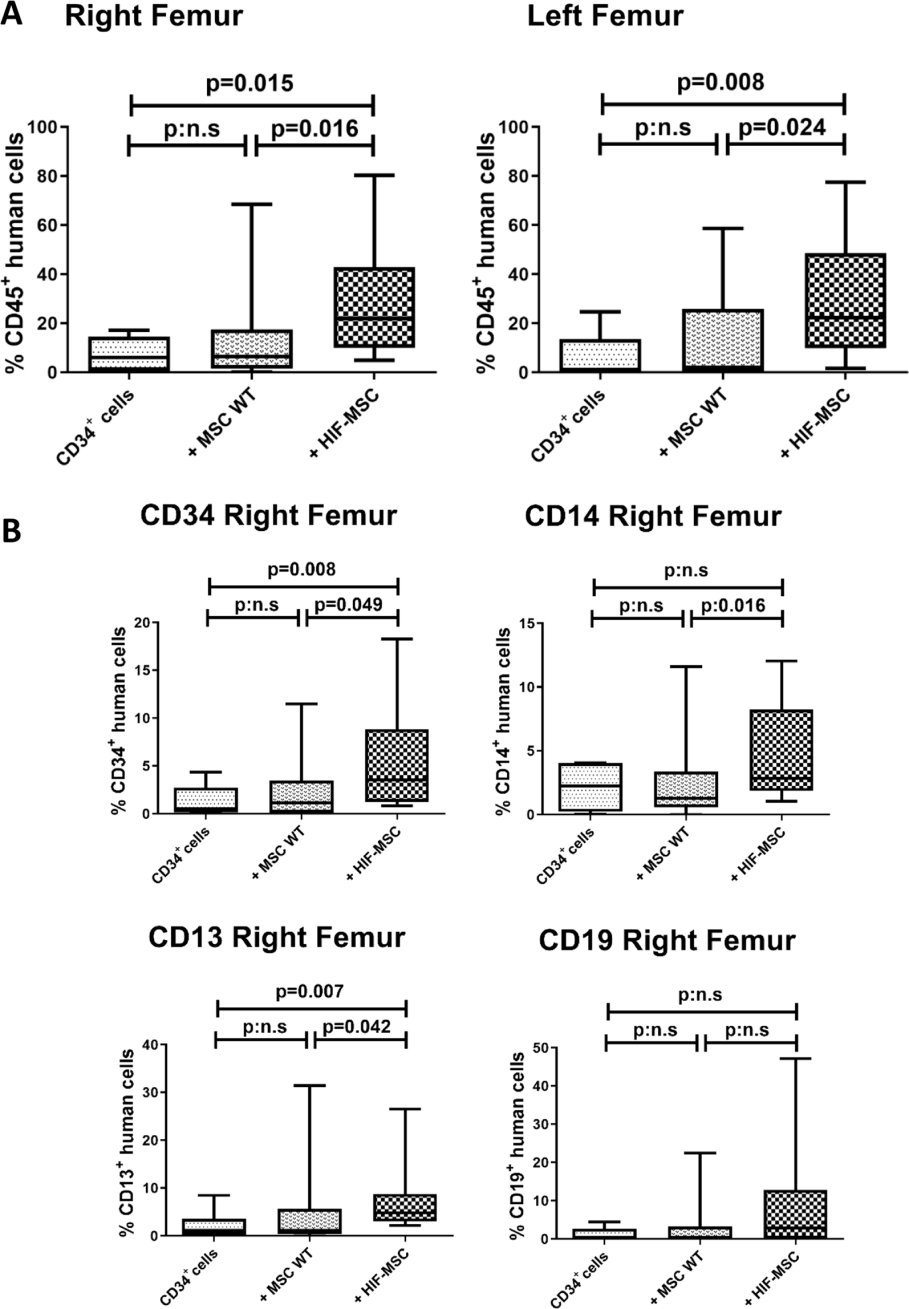
Analysis of human hematopoietic engraftment in vivo. Human hematopoietic engraftment was evaluated by flow cytometry after xenotrasplantation in NOD/SCID mice. The percentage of human CD45^+^ cells (donor chimerism) was analyzed in total bone marrow samples from both femurs, right (MSC injected site) and left (contralateral) 4 weeks after transplantation. Mice were transplanted intravenously with CD34^+^ cells alone or co-transplanted with MSC WT or HIF-MSC, that were administered in the right femur (**A**). Percentage of human hematopoietic subpopulations (CD34, CD14, CD13 and CD19) in the injected femur after 4 weeks was also analyzed (**B**). Data are represented as mean of 8 experiments for mice transplanted with CD34^+^ cells alone, 13 experiments for mice co-transplanted with CD34^+^ cells and WT MSC and 15 experiments for mice transplanted with CD34^+^ cells and HIF-MSC. p:n.s means *p* > 0.05

Finally, the in vivo engraftment ability of CD34^+^ cells was evaluated in a xenotransplantation murine model using NOD/SCID mice assessing the percentage of human CD45^+^ cells after 4 weeks in both femurs. We observed a significant increase in CD34^+^ cell engraftment ability when mice were co-transplanted with HIF-MSC compared to mice that received CD34^+^ or CD34^+^ co-transplanted with MSC WT in both the injected (right) (*p* = 0.016 and *p* = 0.015, respectively) and contralateral (left) femurs (*p* = 0.024 and *p* = 0.008, respectively) (Fig. 3A). Nevertheless, we did not observe significant differences among groups in the spleen (Additional file 4: Figure S3).

Cell engraftment was also evaluated at the subpopulation level assessing CD34^+^, CD14^+^, CD13^+^ and CD19^+^ cell subsets. We observed a significant increase in engraftment of CD34^+^, CD14^+^ and CD13^+^ cell subsets when mice were co-transplanted with HIF-MSC compared with the remaining groups (Fig. 3B). Although the engraftment of the CD19^+^ cell subset was similar to other cell populations, there was no significant differences among experimental groups in our study.

## Discussion

The main originality of the current manuscript is that we have observed, for the first time, that co-transplantation of CD34^+^ cells with HIF-MSC increases their engraftment ability in vivo. In addition, we have described that this may be related to an increased expression of some key molecules involved in engraftment, including CXCR4 and ITGA4 and an improved clonogenic ability induced by MSC in CD34^+^ cells.

HIF-1α overexpression in MSC is not the only modification that has been implemented to enhance MSC properties in different experimental scenarios. For instance, it has been shown that modification of MSC by overexpression of key molecules involved in homing and immunosuppression (as CXCR4 and IL10) or by exofucosylation can improve MSC properties [28–30]. Preconditioning of these cells with pro-inflammatory cytokines, irradiation, pharmacological or chemical agents may have similar effects [31–33]. Our group was one of the firsts to describe that culturing MSC in hypoxia (with 5% O2) improved MSC proliferation and expansion [12]. Several studies have also reported that hypoxia preconditioning enhances the therapeutic potential of MSC [34–38]. However, maintaining in vitro the hypoxic conditions that MSC have physiologically in the human bone marrow niche is difficult to preserve only by regulating culture conditions, since the exposure to a normoxic environment rapidly reverses the gained features induced by hypoxia. There is extensive information about the key role of HIF-1α regulating the hypoxic metabolism [35, 37, 39, 40]. That is why in the current work, we have opted by overexpressing HIF-1α in MSC by lentiviral transduction in order to mimic some of the effects induced by hypoxia and maintain those effects over time.

Delving further into the mechanisms involved in the changes induced in MSC by low oxygen concentrations, it has been shown that the most remarkable is the increase in HIF-1α expression, promoting their regenerative and angiogenic capacity, activating Notch downstream genes, increasing cell proliferation and survival and the retention of stem cell properties as self-renewal and senescence inhibition [35, 37–39, 41]. In previous works, we have observed that most of these changes are also induced when transducing MSC with HIF-1α lentivirus vectors [17, 20–22].

**Fig. 4:**
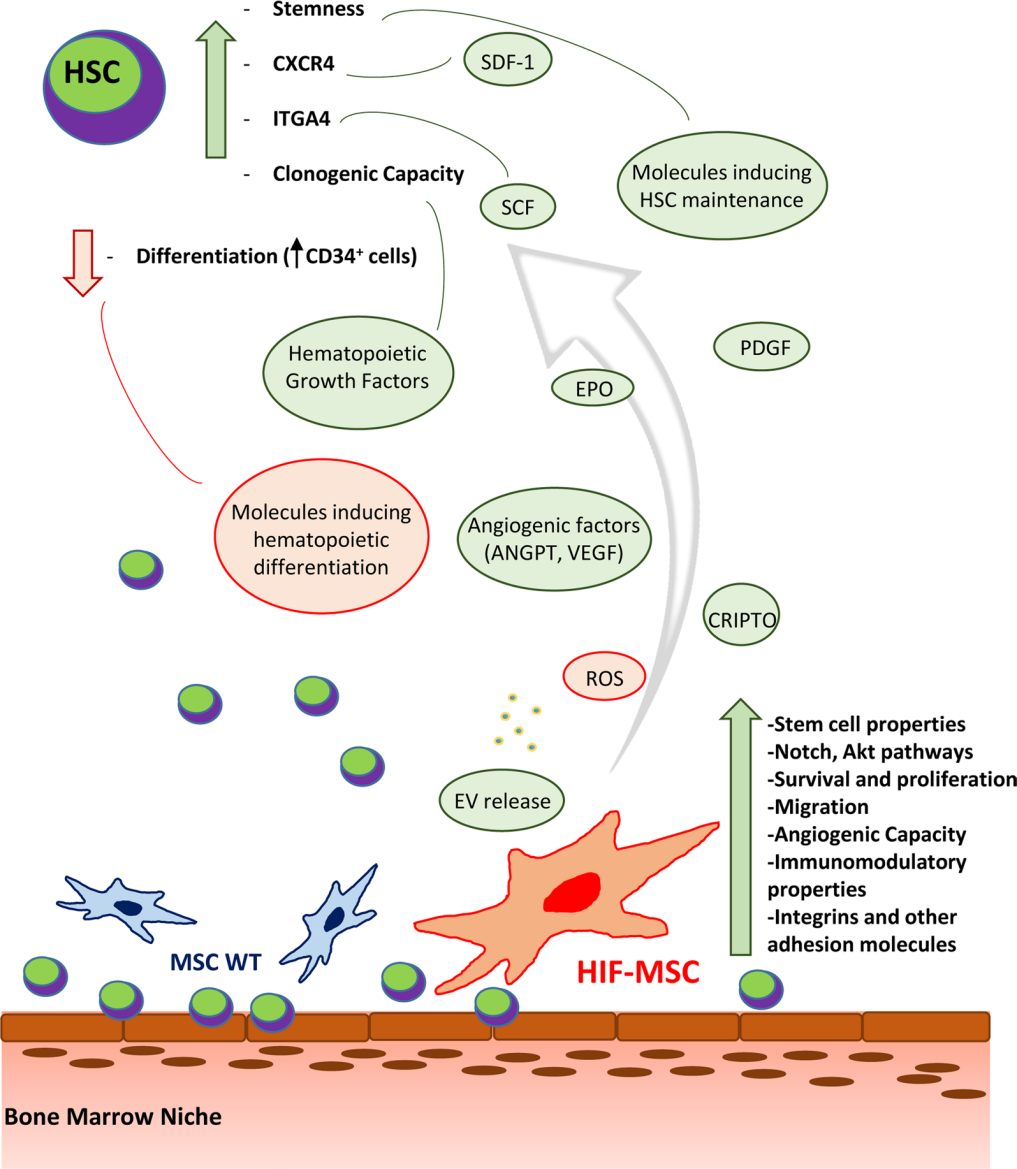
Schematic representation of the potential mechanisms by which HIF-1α overexpression in MSC could enhance CD34^+^ cell engraftment. HIF-1α action may depend more on secreted soluble factors than on cell-to-cell contact. Interaction through ligands and receptors, cytokine release and extracellular vesicle release contribute to this process. Molecules that are increased in HIF-MSC are represented in green and molecules that are decreased are represented in red. HSC, hematopoietic stem cell; CXCR4, C-X-C chemokine receptor type 4; ITGA4, integrin subunit alpha 4; SDF-1, stromal-derived factor-1; SCF, stem cell factor; EPO, erythropoietin; PDGF, platelet-derived growth factor; ANGPT, angiopoietin; VEGF, vascular endothelial growth factor; ROS, reactive oxygen species; EV, extracellular vesicles; MSC WT: MSC wild type; HIF-MSC: MSC overexpressing HIF-1α

Regarding the effects of HIF-1α expression in MSC on the hematopoietic cell compartment that is the focus of our work, Guarnerio et al. demonstrated that downregulation of HIF factors increases interferon expression, which induces expansion and differentiation of hematopoietic progenitors mediated by STAT-1. In fact, during systemic infections, where HSC proliferation and differentiation is required, HIF-1α expression is suppressed in MSC. However, when HIF-1α is overexpressed in MSC they observed a decrease of hematopoietic differentiation while no changes were detected in their proliferation capacity after 4 days of culture [14]. In agreement with these data, we have not seen significant differences regarding viability or cell cycle between CD34^+^ cells co-cultured with HIF-MSC or MSC WT after 3 days. Another report confirms the decrease in hematopoietic differentiation with HIF-MSC but, in contrast, notices an increase in CD34^+^ expansion after 10 days of co-culture [13]. This discordance regarding HSC proliferation may be related to the different experimental conditions used. It seems that HIF factors can regulate hematopoiesis altering MSC secretome, suppressing the release of some molecules that induce hematopoietic differentiation and increasing the release of other factors that induce HSC maintenance, as CRIPTO, a HIF regulated soluble factor [42] (Fig. 4).

ROS signaling is obviously related to hypoxia [43]. In MSC, it has been observed a reduction in ROS when MSC are cultured at low oxygen concentration. Hypoxia induces a stabilization of HIF-1α that leads to anaerobic respiration where less oxygen is consumed and the production of ROS is lowered [35, 37]. We analyzed whether this antioxidant capacity of HIF-1α overexpressed in MSC could influence in any way the ROS status in CD34^+^ cells after co-culture, but we have not observed differences between CD34^+^ cells co-cultured with HIF-MSC or MSC WT.

Several interleukins and paracrine factors are upregulated in hypoxic MSC, together with some genes involved in hematopoiesis and engraftment including vascular endothelial growth factor (VEGF), erythropoietin, angiopoietin, platelet-derived growth factor, CXCR4 or SDF-1 [19, 37, 44, 45]. In this work, we have shown that the expression of CXCR4 in CD34^+^ cells co-cultured with HIF-MSC is significantly increased. This is in accordance with findings that SDF-1 expression, one of the most important ligands of CXCR4, is regulated by HIF-1α in stromal cells increasing homing of CXCR4 positive progenitor cells to reduced oxygen tension areas within the BM niche [46]. SDF-1 expression is directly proportional to tissue oxygen reduction. In fact, MSC overexpressing HIF-1α in our system have also increased expression of SDF-1 [21]. In addition, hypoxia induces an upregulation of CXCR4 in MSC that seems to be driven by HIF-1α [47]. We have not observed changes in c-kit expression in CD34^+^ cells co-cultured with HIF-MSC, although it has been described that the production of its ligand, SCF, is significantly increased when MSC overexpress HIF-1α [13]. SCF also causes upregulation of ITGA4 in CD34^+^ cells [48]. These data are consistent with our findings. ITGA4 expression in HSC co-cultured with HIF-MSC is significantly increased. ITGA4 is an important molecule involved in homing of HSC and is described to be upregulated in hypoxic environments as ischemic tissues or melanoma cells [49, 50]. Expression of other integrins and genes involved in cell adhesion is also upregulated in MSC overexpressing HIF-1α [22]. Additionally, we have detected an increase in CD34 expression in HSC cultured with HIF-MSC, and this could be related to a reduced HSC differentiation, as others have described [13, 14].

Moving to the functional implications of the changes induced in CD34^+^ cells after co-culture with HIF-MSC, we have seen an enhanced clonogenic capacity compared to those co-cultured with MSC WT. In accordance with these results, in another work where HIF-1α was overexpressed in MSC, they observed similar results with an increased number of CD34^+^ colonies after 5 days of co-culture. In this work, they also described that HIF-1α overexpression in MSC can augment the production of some hematopoietic growth factors increasing their hematopoietic supportive functions [13]. This effect could also be mediated by extracellular vesicles (EV). We have previously reported that the incorporation of EV from MSC into CD34^+^ cells increases their clonogenic capacity [25, 51] and other reports showed similar results [52–54]. In fact, HIF-1α action may depend more on secreted soluble factors than on cell-to-cell contact [23]. We have also described that in HIF-MSC, the release of EV is increased and their content is different from EV released by MSC WT [20].

The latest and most relevant result of our study is the significant better engraftment of human CD34^+^ cells co-transplanted with HIF-MSC in comparison with alone or co-transplanted with MSC WT in an established xenotransplantation model. These differences were observed both in the injected and in the contralateral femurs. Although there is no previous published data in this setting, there is an interesting study where extramedullary bone and bone marrow were developed in immmunodeficient mice after injecting subcutaneously human MSC and endothelial cells [55]. They demonstrate human HSC engraftment in this ectopic bone marrow niche, and when HIF-1α was knocked down in MSC, it was significantly reduced. These findings position HIF-1α expression in MSC as a key regulator of their role in favoring human HSC engraftment. We have also evaluated the engraftment of different cell subsets, observing a significant increase of human CD14^+^, CD13^+^ and CD34^+^ cell populations in mice co-transplanted with HIF-MSC. However, no significant improvement of engraftment was observed in the CD19^+^ cells. It has been described that HIF activity needs to be low at the immature B cell stages for a normal B cell development. HIF-1α activation could decrease BCR editing and cause arrest of immature B cells. Administration of HIF activators in clinical use reduce B cells [56], but the effects of co-transplantation of CD34^+^ cells with HIF-MSC on B cell development remains to be elucidated. Regarding potential mechanisms for these enhanced effects of modified MSC, in previous works we have described that overexpression of HIF-1α increases MSC migration ability and their cell adhesion capacity after intravenous administration, one of the limiting factors in the therapeutic potential of MSC [22]. We have also observed an improvement in their immunomodulatory capacity [57, 58]. Moreover, their osteogenic differentiation ability has been shown to be increased in MSC overexpressing HIF-1α [59]. All these data support the improvement of HSC engraftment after MSC modification. It would have been of interest the evaluation of CXCR4 and ITGA4 expression in the engrafted cells at 4 four weeks postinjection to examine if mice with higher human chimerism display a higher expression of these molecules. There is a study including patients undergoing HSCT where the authors found significant differences in CXCR4 expression between patients undergoing successful engraftment and those with poor or no engraftment at day 90 [60]. This evaluation would allow to assess, using the CXCR4 inhibitor AMD3100, if the effect of HIF-1α can be diminished through the inhibition of the SDF-1-CXCR4 axis.

## Conclusions

In summary, we have described that overexpression of HIF-1α in MSC increases their favorable effects on supporting human CD34^+^ cell engraftment in vivo. This is also associated with an increase in the clonogenic capacity of hematopoietic cells and changes in adhesion molecules involved in these processes that are induced after the co-culture with modified MSC.

## Supplementary Information

**Additional file 1**. Additional methods.

**Additional file 2. Figure S1**. Transduction efficiency of MSC. Histogram of a representative sample, with transfection efficiency >90%. MSC WT: MSC wild type; HIF-MSC: MSC overexpressing HIF-1α; GFP: Green Fluorescent Protein

**Additional file 3. Figure S2**. Immunophenotypic analysis of MSC WT and HIF-MSC. Histogram of the different surface molecules analyzed by flow cytometry. MSC WT: MSC wild type; HIF-MSC: MSC overexpressing HIF-1α

**Additional file 4. Figure S3**. Analysis of human hematopoietic engraftment in spleen. Human hematopoietic engraftment was evaluated by flow cytometry after xenotrasplantation in NOD/SCID mice. The percentage of human CD45^+^ cells (donor chimerism) was analyzed in total spleen samples 4 weeks after transplantation. Mice were transplanted intravenously with CD34^+^ cells alone or co-transplanted with MSC WT or HIF-MSC, that were administered in the right femur. Data are represented as mean of 8 experiments for mice transplanted with CD34^+^ cells alone, 13 experiments for mice co-transplanted with CD34^+^ cells and WT MSC and 15 experiments for mice transplanted with CD34^+^ cells and HIF-MSC. p:n.s means p > 0.05

## Data Availability

The datasets supporting the conclusions of this article are available from the corresponding author upon reasonable request.

## Abbreviations

allo-HSCT: Allogeneic hematopoietic stem cell transplantation
HSC: Hematopoietic stem cells
BM: Bone marrow
MSC: Mesenchymal stromal cells
GVHD: Graft-versus-host disease
HIF-1α: Hypoxia-inducible factor-1α
DMEM: Dulbecco’s modified Eagle’s medium
FBS: Fetal bovine serum
UCB: Umbilical cord blood
7-AAD: 7-Amino-actinomycin D
MSC WT: MSC wild type
HIF-MSC: MSC overexpressing HIF-1α
RPMI: Roswell Park Memorial Institute Medium
ROS: Reactive oxygen species
DCFH-DA: Dichlorodihydrofluorescein diacetate
MFI: Median fluorescence intensity
PBS: Phosphate-buffered saline
SCF: Stem cell factor
GM-CSF: Granulomonocytic colony-stimulating factor
G-CSF: Granulocytic colony-stimulating factor
CFU-GM: Colony-forming unit granulocyte/macrophage
NOD/SCID: Nonobese diabetic/severe combined immunodeficient mice
CXCR4: C-X-C motif chemokine receptor 4
ITGA4: Integrin subunit alpha
VEGF: Vascular endothelial growth factor
EV: Extracellular vesicles
