# Supplementary material for: Co-administration of human MSC overexpressing HIF-1α increases human CD34+ cell engraftment in vivo
Source: Stem Cell Res Ther. 2021 Dec 7;12:601. doi: 10.1186/s13287-021-02669-z (PMC8650423; doi:10.1186/s13287-021-02669-z)
Supplement: Supplementary file 1 — Additional file 1. Additional methods. [file 13287_2021_2669_MOESM1_ESM.docx]

**SUPPLEMENTARY METHODS**

**FLOW CYTOMETRIC ANALYSIS FOR MSC CHARACTERIZATION**

MSC were incubated with the following monoclonal antibodies: PE-conjugated CD166, PCy7-conjugated CD90, APC-conjugated CD44, APCH7-conjugated CD73, BV421-conjugated CD105, BV510-conjugated CD45 and HLA-DR and 7-AAD. CD166 and 7-AAD were purchased from BD Biosciences, CD44, CD105, CD73, CD45 and HLA-DR from Biolegend, CD90 and CD34 from Invitrogen and CD14 from cytognos.

**FLOW CYTOMETRIC ANALYSIS OF PROTEINS INVOLVED IN HEMATOPOIESIS**

CD34^+^ cells were incubated with the following monoclonal antibodies: PE-conjugated CD34, APC-conjugated CD184, PC7-conjugated CD117, APCH7-conjugated CD49d and 7-AAD. All of them were purchased from BD Biosciences (San José, CA) except for CD117 (Beckman Coulter).

**ANALYSIS OF HUMAN HEMATOPOIETIC ENGRAFTMENT BY FLOW CYTOMETRY**

For flow cytometry studies, bone marrow samples were obtained from both femurs. All cell suspensions were collected in PBS, red blood cells were lysed ~~lysated~~ by treatment with ammonium chloride and then resuspended again in PBS. To analyze human hematopoietic cell engraftment, samples were stained with FITC-conjugated anti-human CD45 (Immunostep, Salamanca Spain). For the analysis of the engraftment of different hematopoietic subpopulations, cells were stained with PE-conjugated CD13, APC-conjugated CD19, PE-conjugated CD14 and APC-conjugated CD34 (Cytognos, Salamanca, Spain), all of them anti-human. PerCP-Cy5.5-conjugated anti-mouse CD45 was used in order to verify the specificity of anti-human CD45 for human cells. In all cases, samples were also stained with 7-amino-actinomycin D (7-AAD; Becton Dickinson Biosciences). Besides, an unstained control was included for each sample. For data analysis, CD45 expression was used to gate human cells and 7-AAD to exclude dead cells. Then, human hematopoietic subpopulations were detected by CD13 (myeloid cells), CD14 (monocytes), CD34 (progenitor hematopoietic cells) and CD19 (B-cells) expression within the human CD45^+^ live gate.
