# Supplementary figures and images for: Co-administration of human MSC overexpressing HIF-1α increases human CD34+ cell engraftment in vivo
Source: Stem Cell Res Ther. 2021 Dec 7;12:601. doi: 10.1186/s13287-021-02669-z (PMC8650423; doi:10.1186/s13287-021-02669-z)

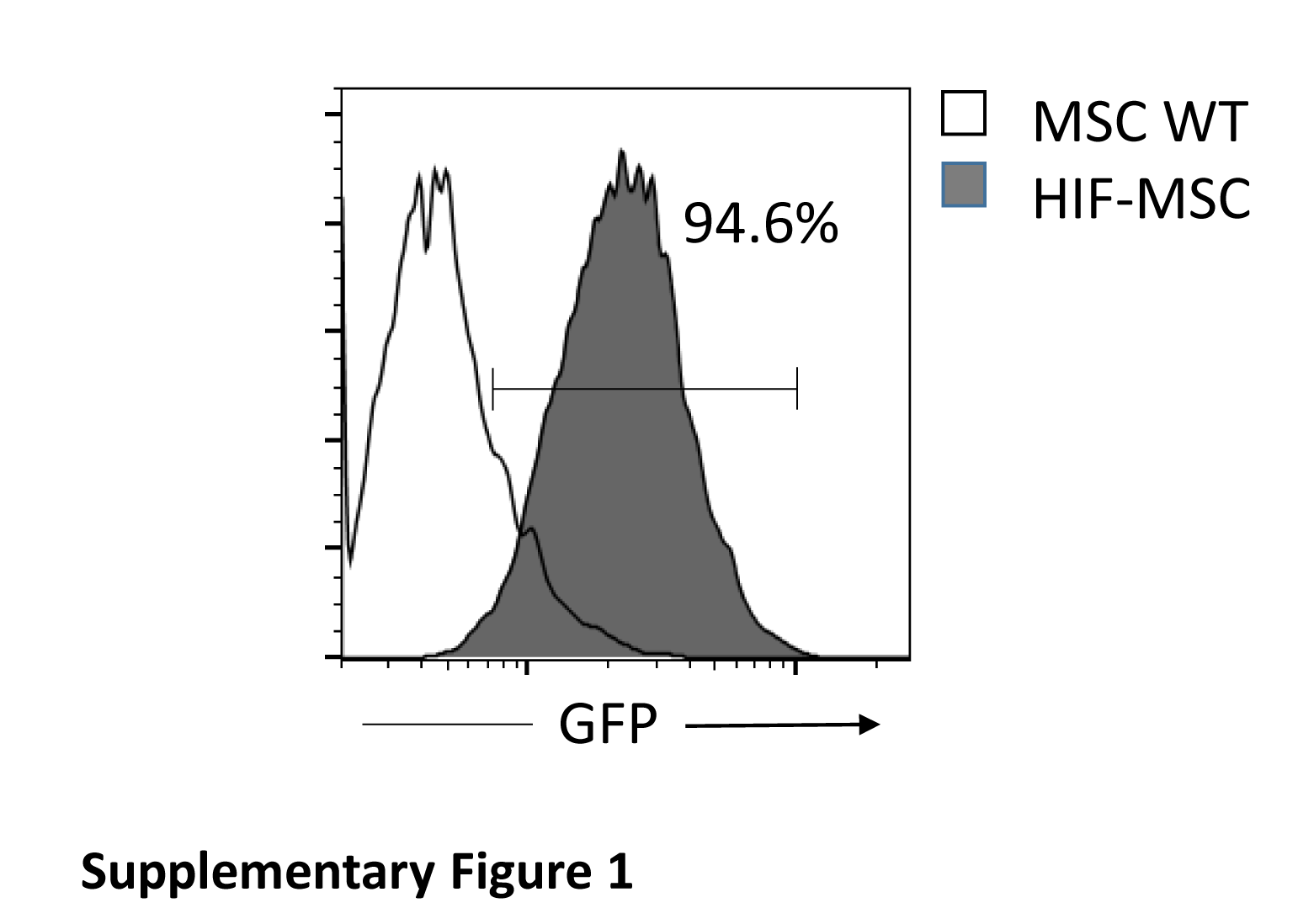

Supplement: Supplementary file 2 — Additional file 2. Figure S1. Transduction efficiency of MSC. Histogram of a representative sample, with transfection efficiency >90%. MSC WT: MSC wild type; HIF-MSC: MSC overexpressing HIF-1α; GFP: Green Fluorescent Protein [file 13287_2021_2669_MOESM2_ESM.tif]

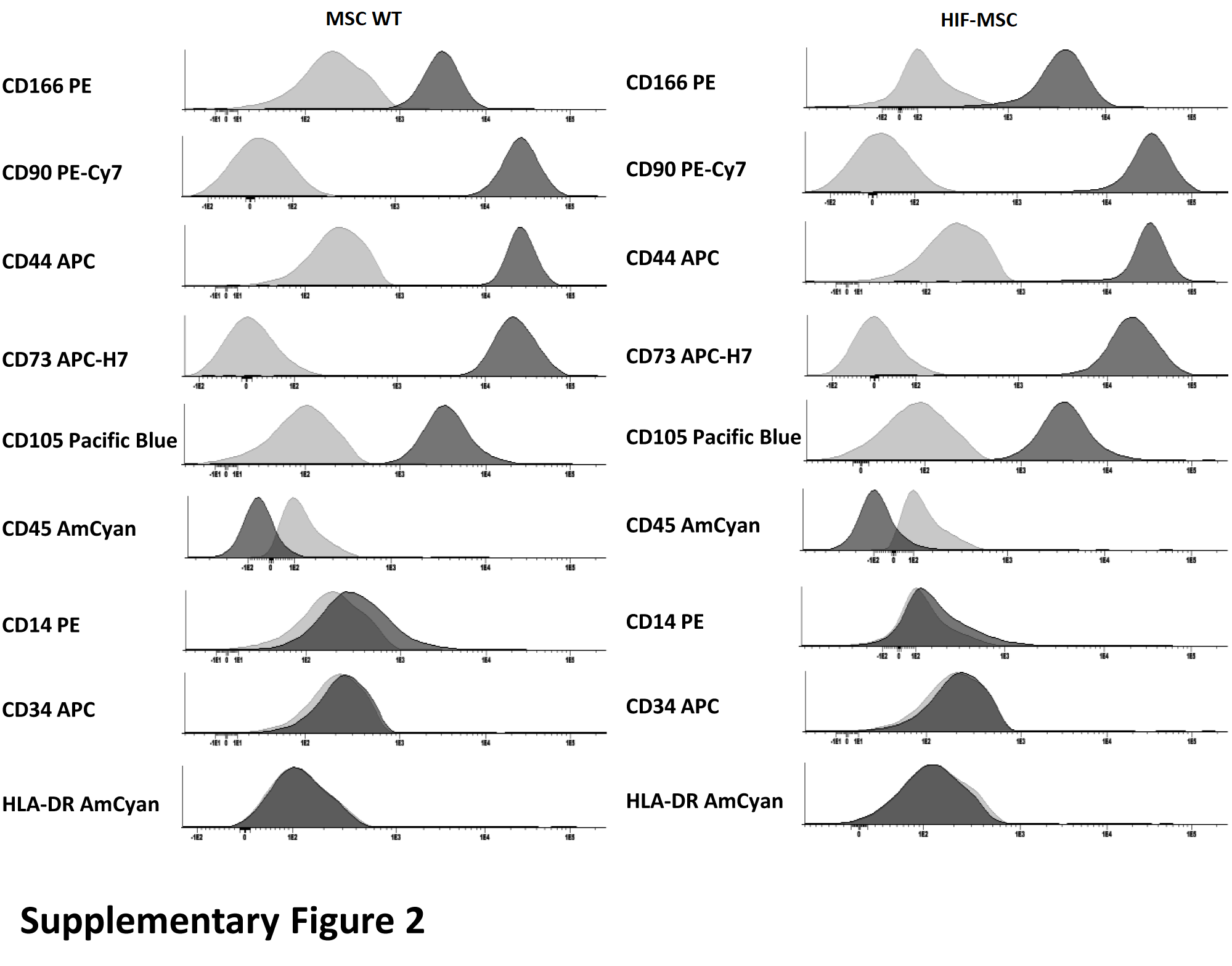

Supplement: Supplementary file 3 — Additional file 3. Figure S2. Immunophenotypic analysis of MSC WT and HIF-MSC. Histogram of the different surface molecules analyzed by flow cytometry. MSC WT: MSC wild type; HIF-MSC: MSC overexpressing HIF-1α [file 13287_2021_2669_MOESM3_ESM.tif]

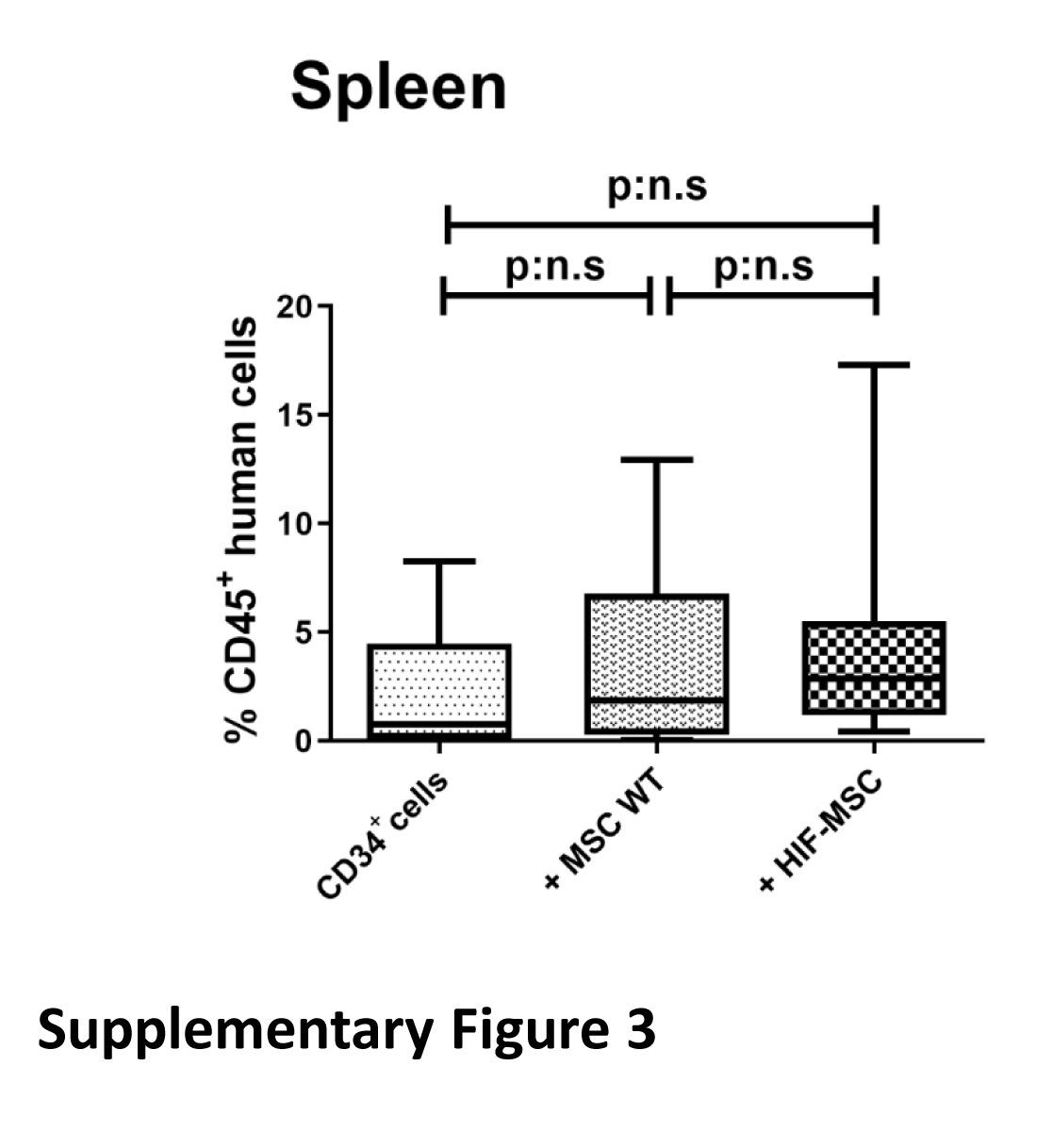

Supplement: Supplementary file 4 — Additional file 4. Figure S3. Analysis of human hematopoietic engraftment in spleen. Human hematopoietic engraftment was evaluated by flow cytometry after xenotrasplantation in NOD/SCID mice. The percentage of human CD45+ cells (donor chimerism) was analyzed in total spleen samples 4 weeks after transplantation. Mice were transplanted intravenously with CD34+ cells alone or co-transplanted with MSC WT or HIF-MSC, that were administered in the right femur. Data are represented as mean of 8 experiments for mice transplanted with CD34+ cells alone, 13 experiments for mice co-transplanted with CD34+ cells and WT MSC and 15 experiments for mice transplanted with CD34+ cells and HIF-MSC. p:n.s means p > 0.05 [file 13287_2021_2669_MOESM4_ESM.tif]
